# Intra-Cranial and Extra-Cranial Hæmorrhages in the New-Born

**Published:** 1904-05-14

**Authors:** 


### INTRA-CRANIAL AND EXTRA-CRANIAL HÆMORRHAGES IN THE NEW-BORN.

Mr. J. Howell Evans[1] puts forward a theory according to which cephalhæmatoma externa and intra-cranial hæmorrhage in the new-born are attributable to a common origin. He points out that these hæmorrhages occur independently of many factors which have commonly been considered as the causes of their production; they arise irrespective of the lie and position of the fœtus in utero, without any relation to the nature of the presentation, and in labours unaided by forceps, as well as in labours where forceps have been used. Mr. Evans believes that the over-riding of the bones of the skull, during moulding of the fœtal head at birth, is the chief factor. In the adaptation of the child's skull to the maternal pelvis, one parietal bone overlaps the other at the sagittal suture, while at the coronal and lambdoid sutures both parietal bones over-ride the frontal and occipital bones. If this moulding involves certain accessory sutures, the blood-vessels which traverse these accessory sutures may be injured; and it is to such an occurrence that Mr. Evans attributes the hæmorrhage. The parietal bone, he points out, may be developed from two centres, in which case a gap exists for some time at the spot where the bones derived from the two parietal centres of each side approach each other. This space is usually closed in about the fourth month of intra-uterine life. It may, on the other hand, persist until birth, and a trace of it may remain through life as the parietal foramen. Transmitted through the parietal foramen, intra-parietal suture, or sagittal fontanelle, as the condition may be, are blood-vessels. If then the over-riding of the bones of the vault takes place at the intra-parietal suture as it does at the other sutures, it is clear that these vessels may be nipped and hæmorrhage caused either between the outer surface of the bone and its periosteum, or inside the skull between the parietal bone and the dura mater.

[1] Brit. Jour. Child. Dis., May.

